# Multiallelic copy number variation in the complement component 4A (*C4A*) gene is associated with late-stage age-related macular degeneration (AMD)

**DOI:** 10.1186/s12974-016-0548-0

**Published:** 2016-04-18

**Authors:** Felix Grassmann, Stuart Cantsilieris, Anja-Sabrina Schulz-Kuhnt, Stefan J. White, Andrea J Richardson, Alex W Hewitt, Brendan J. Vote, Denise Schmied, Robyn H Guymer, Bernhard H.F. Weber, Paul N. Baird

**Affiliations:** Institute of Human Genetics, University of Regensburg, Regensburg, 93053 Germany; Centre for Eye Research, University of Melbourne, Royal Victorian Eye and Ear Hospital, Melbourne, Australia; School of Medicine, Menzies Research Institute Tasmania, University of Tasmania, Hobart, Tasmania Australia; Leiden University Medical Center, Leiden, The Netherlands

**Keywords:** Age-related macular degeneration, AMD, *C4*, Complement, Copy number variation (CNV), Multiallelic, Genetic association

## Abstract

**Background:**

Age-related macular degeneration (AMD) is the leading cause of vision loss in Western societies with a strong genetic component. Candidate gene studies as well as genome-wide association studies strongly implicated genetic variations in complement genes to be involved in disease risk. So far, no association of AMD with complement component 4 (*C4*) was reported probably due to the complex nature of the *C4 *locus on chromosome 6.

**Methods:**

We used multiplex ligation-dependent probe amplification (MLPA) to determine the copy number of the *C4 *gene as well as of both relevant isoforms, *C4A *and *C4B*, and assessed their association with AMD using logistic regression models.

**Results:**

Here, we report on the analysis of 2645 individuals (1536 probands and 1109 unaffected controls), across three different centers, for multiallelic copy number variation (CNV) at the *C4 *locus. We find strong statistical significance for association of increased copy number of *C4A *(OR 0.81 (0.73; 0.89);*P* = 4.4 × 10^−5^), with the effect most pronounced in individuals over 78 years (OR 0.67 (0.55; 0.81)) and females (OR 0.77 (0.68; 0.87)). Furthermore, this association is independent of known AMD-associated risk variants in the nearby *CFB/C2 *locus, particularly in females and in individuals over 78 years.

**Conclusions:**

Our data strengthen the notion that complement dysregulation plays a crucial role in AMD etiology, an important finding for early intervention strategies and future therapeutics. In addition, for the first time, we provide evidence that multiallelic CNVs are associated with AMD pathology.

**Electronic supplementary material:**

The online version of this article (doi:10.1186/s12974-016-0548-0) contains supplementary material, which is available to authorized users.

## Background

Age-related macular degeneration (AMD) is the leading cause of severe vision loss in aging societies [1–3]. An early sign of AMD is the appearance of the so-called drusen, which are yellowish extracellular deposits of protein and lipid material within and beneath the retinal pigment epithelium (RPE). Late-stage AMD can manifest essentially as two distinct forms—geographic atrophy (GA) and neovascular (NV) AMD, with both late-stage forms in a proportion of cases presenting in the same or in different eyes of an individual. GA occurs in up to 50 % of cases and is clinically defined as a distinct area of RPE cell atrophy with slow progression over the years. NV AMD describes the growth of blood vessels beneath and within the retina and is mostly characterized by hemorrhagic detachment of the RPE or the retina and eventually widespread RPE atrophy. Progression to visual loss can be rapid in NV AMD [4].

Over the past several years, genome-wide association studies and large scale re-sequencing projects have identified a number of single nucleotide variants (SNVs) enriched in complement and complement-related genes that confer a strong risk for AMD [3, 5–8]. Recently, a genome-wide association study conducted by the International AMD Genomics consortium (IAMDGC) identified 52 independent genetic signals at 34 loci to be associated with AMD risk [9], explaining around half of the genomic heritability of the disease. Six out of those 34 loci harbor one or more complement or complement-related genes.

Assessment of structural variation, particularly in duplicated regions of the genome, and its contribution to disease still remains a challenge for most case-control studies [10, 11]. Multiallelic loci are especially problematic, due to a lack of suitable tagging variants and a dearth of probes on commercial microarrays in duplication rich regions of the genome [12]. We and others have previously demonstrated that multiallelic loci can be genotyped economically and accurately using PCR-based quantitative techniques [13–15]. Here, we applied multiplex ligation-dependent probe amplification (MLPA) [16] to examine the role of the multiallelic complement component 4 (*C4*) copy number variations (CNVs) as part of the classical complement pathway in AMD etiology.

In this study, we successfully genotyped CNVs for *C4 *and its relevant isoforms (*C4A*,* C4B*) in 2645 individuals from three large AMD cohorts from Australia and Germany. We identify strong statistical significance for a protective association of *C4A *copy number and AMD. Stratification of individuals based on age and gender revealed that the protective effect increases with increasing age and is stronger in females. In addition, this association appears to be independent of other, strongly associated AMD variants at the nearby *CFB/C2 *locus. These data implicate multiallelic CNV of the *C4 *locus in AMD susceptibility, providing further evidence for the crucial role of complement dysregulation in AMD etiology.

## Methods

### Subjects

Three independent patient cohorts were included in our study comprising a total of 1536 unrelated Caucasian patients with clinically documented late-stage AMD (cases) and 1109 unrelated individuals with comparable age range and ethnicity without signs of macular disease (controls). The controls were often spouses of AMD patients (Table 1). Cases and controls were examined by trained ophthalmologists. Fundus photographs were graded according to standardized classification systems as described previously [17, 18]. The study was conducted at all sites in strict adherence to the tenets of the Declaration of Helsinki and was approved by the respective Ethics Committees of the Human Research and Ethics Committee of the Royal Victorian Eye and Ear Hospital (RVEEH), Melbourne, the University of Tasmania, Australia and at the University Eye Clinics of Würzburg, München and Tübingen, Germany.

**Table 1:** Summary characteristics of participating studies

	Number of individuals						Mean age (years) (SD)		Females	
Study	Cases	GA cases	NV cases	GA and NV cases	Controls	Total	Cases	Controls	Cases (%)	Controls (%)
AUS study	633	137	496	0	404	1037	75.95 (8.50)	71.03 (6.92)	64.60	55.40
WUE study	547	220	287	40	384	931	73.54 (6.74)	78.26 (5.30)	63.90	63.02
MUE/TUE study	356	97	129	130	321	677	76.16 (7.54)	69.50 (8.25)	64.89	55.80
Full study	1536	454	912	170	1109	2645	75.14 (7.78)	73.09 (7.83)	64.45	58.15

*GA *geographic atrophy, *NV* neovascular disease, *SD *standard deviation, *AUS *Australian study, *WUE/MUE/TUE *German study

### Genotyping by MLPA

Genomic DNA was extracted from peripheral blood leukocytes according to established protocols. MLPA was performed according to previous reports [19, 20] with custom-made oligonucleotide probes (Additional file 1: Table S1). For each PCR reaction, 1 μl was mixed with 8.8 μl of HIDI formamide and 0.2 μl of LIZ500 size standard (Applied Biosystems). PCR products were separated by capillary electrophoresis on a sequence platform (3130xl Genetic Analyzer, Applied Biosystems; Foster City, CA, USA). The program Peak Scanner 2 (Applied Biosystems) was used to extract the dosages (height) of each peak. Copy number analysis was performed by dividing the peak height of each test probe (*C4A*, *C4B*, or *C4*-ex30 representing the isoforms *C4A *and *C4B *or the total *C4 *copy number, respectively), by the sum peak heights from two control loci (*EP300 *and *CREBBP*) to generate a normalized ratio. The normalized ratio was then divided by the median of all ratios for the corresponding peak, yielding a normalized probe dosage. For the AUS study, the *C4*-ex30 probe failed to produce a product. For those samples, total *C4 *dosage was calculated from the mean of both *C4A *and *C4B* dosages. We show that this approach is feasible as there is a strong correlation between total *C4 *dosage and the mean of both, *C4A *and *C4B *dosage in the WUE and MUE/TUE studies (Additional file 2: Figure S1). The normalized probe dosages were ordered from low to high, and subgroups corresponding to different DNA copy numbers were calculated by determining the relative differences between the subgroups. The distribution of copy numbers of *C4A*, *C4B*, and total *C4 *in our controls was comparable to previously reported distributions in healthy individuals (Additional file 3: Table S2).

### Direct PCR of C4 isoforms

To confirm homozygous deletions of *C4A *and *C4B*, we performed a duplex PCR with two sets of isotype-specific primers as described previously [21]. In brief, PCRs were performed using Qiagen Hotstart Reagents (www.qiagen.com) and oligonucleotide primers as listed in Additional file 4: Table S3. The presence or absence of the *C4A *and *C4B *genes was assessed in 2 % agarose gels.

### Genotyping of AMD-associated variations in the CFB/C2 locus

Several previously reported AMD risk-associated variations in *CFB *and *C2 *are close to the *C4 *gene (a distance of approximately 30,000 bp). Thus, known risk-associated haplotypes carrying known AMD-associated variations can potentially drive the association of *C4 *CNV. Recently, a GWAS conducted by the International AMD Genomics Consortium (IAMGDC) implicated four independent signals in this locus to be associated with AMD [9]. We therefore determined the genotypes of four risk variants to represent the four associated signals (termed independent hit 8.1 to 8.4 in [9]). Two thousand two hundred fifty-one DNA probes were genotyped on the HumanCoreExome Chip (Illumina) as part of the IAMDGC study. For those samples, we extracted the genotypes of rs429608 (8.1), rs114190211 (8.2), rs204993 (8.3), and rs142511358 (8.4) and fit multivariate logistic regression models additionally conditioned on those four variants.

To further characterize the CFB/C2 locus, we used SHAPEIT2 [22] with standard settings to phase the genotypes and obtain the haplotypes. The association of the resulting haplotypes with AMD risk was then analyzed in a multivariate logistic regression model adjusted for age, gender, and study. In addition, since the phase of the CNVs is difficult to estimate, we calculated the average number of copies present on each haplotype to identify haplotypes that carry C4A CNVs.

### Statistical analysis and visualization

Copy number association analysis was carried out by logistic regression adjusted for age and gender and, where appropriate, additionally adjusted for study [17]. All analyses modeled an additive genetic effect, and the genotype was coded as the number of gene copies present at *C4A*, *C4B*, or total *C4*. As *C4 *copy number was analyzed in three independent studies, we subsequently combined the obtained effect sizes (log odds ratios) and standard errors from the three independent studies by conducting a meta-analysis assuming a random effects model. We also assessed the evidence for possible heterogeneity between the estimates from each study by computing the *I*^2^ measure [23]. In the association analyses, the obtained *P* values were adjusted by a conservative Bonferroni correction to account for multiple testing assuming three independent tests due to three CNVs analyzed.

We also performed association testing in groups of individuals stratified by age, gender, and disease subtype (Additional file 5: Table S4 and Additional file 6: Figure S2). The disease subtype-specific analyses compared copy numbers of AMD patients with GA or NV or mixed GA and NV to the copy numbers found in all controls.

To account for possible differences in the association between age groups, we stratified our case-control study into three age categories with roughly equal sample size (1/3 of the total sample size). Accordingly, the samples were assigned either to the youngest group (<71 years old, 918 individuals), to the middle age group (older than 71 years but younger than 78 years, 881 individuals), or to the oldest group (older than 78 years, 840 individuals). Similarly, cases and controls were stratified by their gender and analyzed.

To assess whether the association of *C4 A*CNV with AMD is independent of known AMD-associated signals in this region, we fit logistic regression models additionally conditioned on four AMD-associated SNPs at the *C2/CFB *locus (rs429608, rs114190211, rs204993, and rs142511358). Furthermore, the association of haplotypes reconstructed from variations in the *C2/CFB *locus were stratified by age and gender, adjusted for age and study. The number of samples included in each analysis is indicated by the size of the rectangles in Additional file 6: Figure S2 and Additional file 7: Figure S3.

## Results

### Assessment of C4 gene copy number

**Fig. 1:**
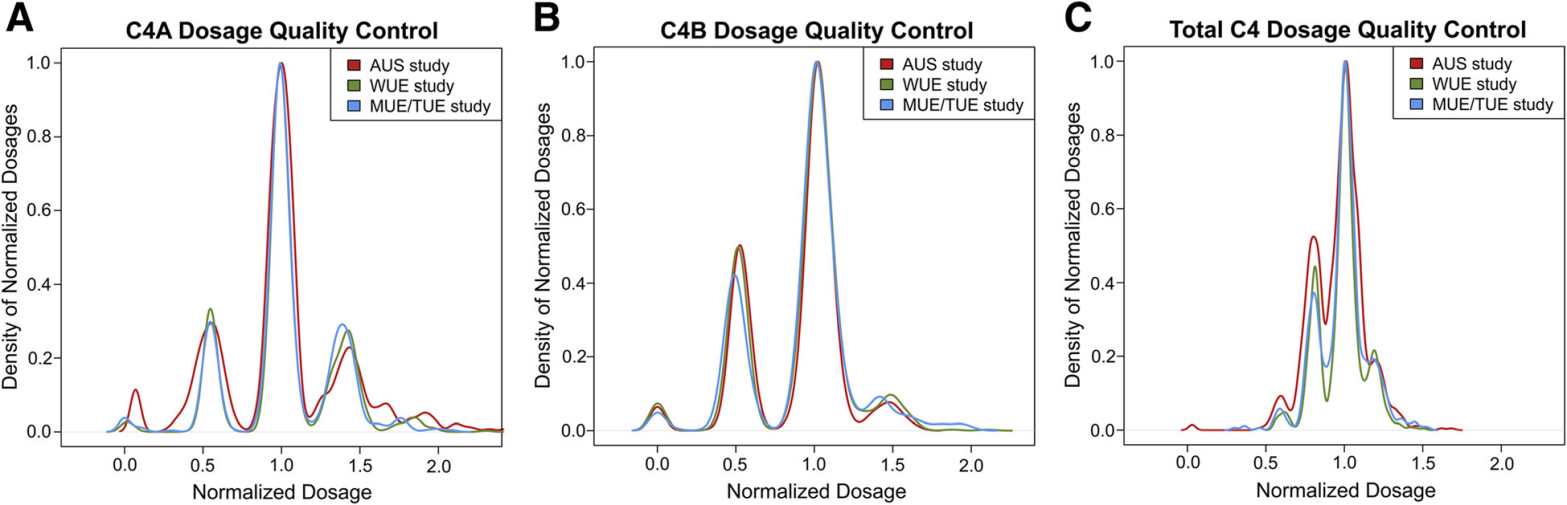
Normalized probe dosage quality control of complement *C4A *(**a**),*C4B *(**b**), and total *C4 *(**c**) in three independent studies. Distribution of unrounded MLPA-based dosage estimates for 2645 individuals from three studies (AUS, WUE, and MUE/TUE) are shown. Distinct peaks corresponding to integer copy numbers are demonstrated for *C4A*, *C4B*, and total *C4*. There is no obvious discrepancy in the distribution of the normalized dosages between studies

The multiallelic CNV at the*C4*locus was assessed by MLPA in three independent studies totalling 1536 late-stage AMD cases of European descent and 1109 age and ethnicity-matched AMD-free controls (Table 1). The published MLPA probe sequences [24] were adapted to facilitate the parallel assessment of the multiallelic CNV for*C4*and its two isoforms *C4A *and *C4B*, despite the two isoforms sharing >99 % sequence identity [25, 26] (Additional file 1: Table S1). We observed that the range of copy numbers in control individuals was comparable to distributions observed in previous studies of European descent (Additional file 3: Table S2) [27, 28] and that the distribution of copy number integers matched those reported previously (*C4A *between 0 and 5 and *C4B *between 0 and 4) [29]. A comparison between cohorts revealed a distinct clustering of results around several maxima, corresponding to integer copy numbers (Fig. 1). As homozygous deletions can be miscalled due to unexpected polymorphism at the site of ligation between two MLPA probes [16], we confirmed all suspected homozygous deletions by direct isotype PCR as previously described [21] and achieved 100 % concordance. It has been suggested that batch effects could result in differential bias leading to false-positive associations in quantitative data sets seeking to measure complex multiallelic CNVs [13]. We therefore examined the distribution of raw unrounded probe dosages for diploid copy number carriers for both *C4A *and *C4B *or tetraploid copy number carriers for total C4 in cases and controls (Additional file 8: Table S5 and Fig. 1). We found no evidence that mean distributions were significantly different between cases and controls, indicating that association results should represent true biological signals.

### Association of C4 CNVs with AMD risk

**Fig. 2:**
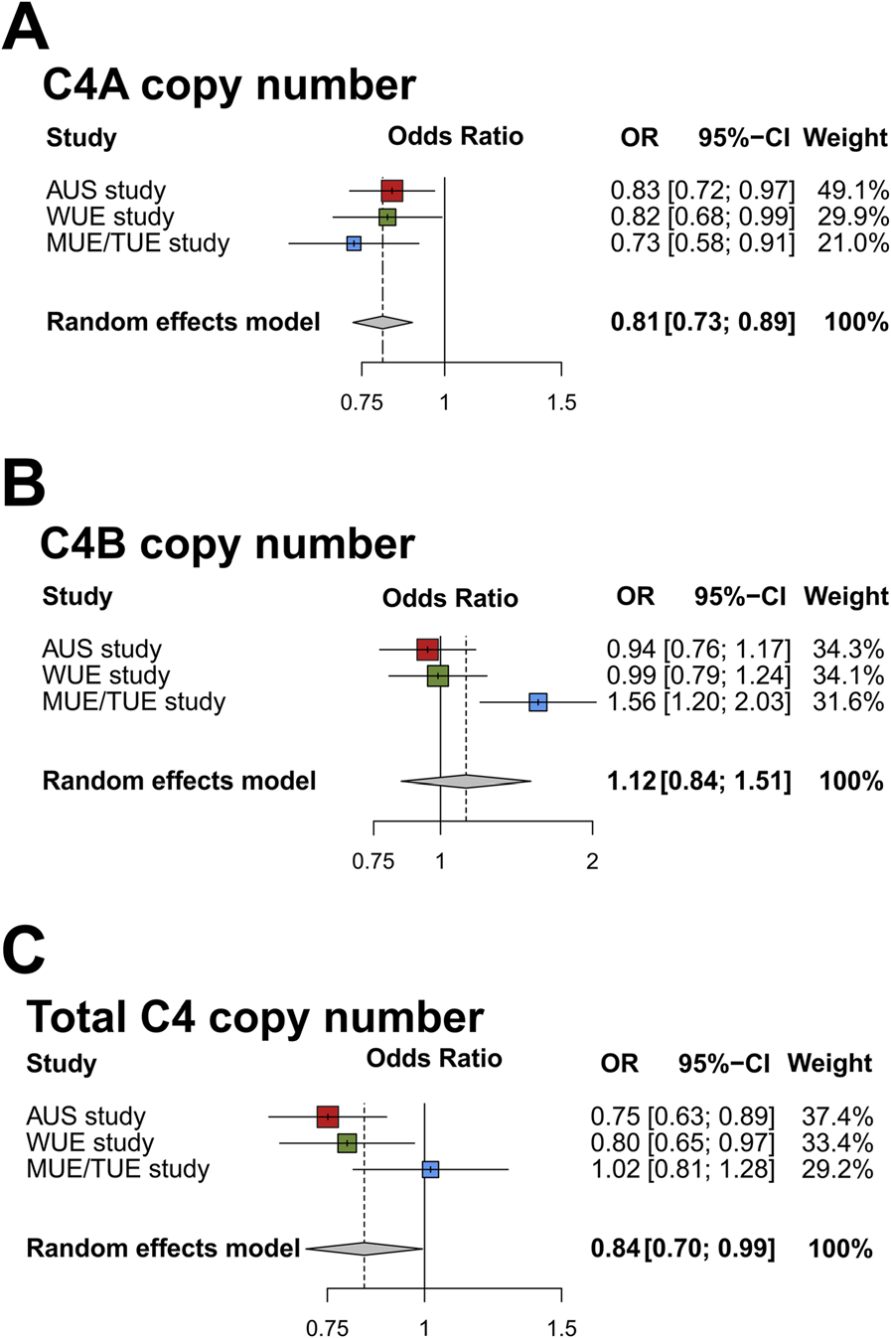
Association analysis of multiallelic complement *C4A *(**a**), *C4B *(**b**), and total *C4 *(**c**) copy numbers in AMD. Multivariate logistic regression models, adjusted for age and gender, were fitted for *C4A*, *C4B*, and total *C4 *copy number in each study. Log odds ratios and standard errors obtained from each study were combined and a meta-analysis performed assuming a random effects model. The combined estimates for the odds ratios and 95 % confidence intervals (CI) were computed from the random effects model. For C4B CNV, there was statistically significant evidence for heterogeneity of the effect sizes between the studies (*P*_heterogeneity_ < 0.05)

Logistic regression models adjusted for age and gender were computed for each study separately, and meta-analyses of effect sizes and standard errors were subsequently conducted assuming a random effects model (Fig. 2). While there was no statistically significant association for multiallelic CNVs at *C4B *or total *C4 *with AMD (*P* > 0.05), we identified a statistically significant association of*C4A*copy number with AMD risk in each study (*P*_uncorrected_ < 0.05, Fig. 2). In the combined study and after correcting for multiple testing, there was a highly significant association of *C4A *copy number with AMD (*P* = 4.4 × 10^−5^,*P*_corrected_ = 1.3 × 10^−4^, Fig. 2). We observed odds ratios (ORs) lower than one per *C4A *copy, indicating a protective effect for each additional *C4A *copy (OR 0.81 (0.73; 0.89)). In a sensitivity analysis, *C4A *copy numbers from each independent study were pooled and analyzed jointly. An unadjusted model was fitted for *C4A *association (Additional file 5: Table S4) identifying statistical significance with AMD in the pooled study (OR 0.83 (0.76; 0.92)). We also computed a logistic regression model adjusted jointly for age, gender, and study and found ORs similar to the unadjusted model (OR 0.82 (0.74; 0.90)) (Additional file 5: Table S4).

### Sensitivity analysis of the observed C4A association

Next, we fitted logistic regression models to groups of individuals stratified by age, gender, or disease phenotype adjusted for study. While no striking differences were found between the three late-stage AMD disease subtypes of only geographic atrophy (GA), only neovascular AMD (NV) or both late-stage forms (GA and NV), a striking difference between age groups and between gender became apparent (Additional file 5: Table S4). The observed protective association was stronger (more protective per *C4A *copy) in individuals older than 78 years (OR 0. 67 (0.55; 0.81)) compared to individuals between 71 and 78 years (OR 0.82 (0.74; 0.90)) and individuals younger than 71 years (OR 0.94 (0.79; 1.12)). Of note, although we did not find a statistically significant difference between the age groups (*P* > 0.05), this effect was present in each study individually. The observation that the strength of the protective effect increases with age is rather unexpected, as genetic associations tend to show stronger effect sizes for adverse and protective alleles in younger individuals [30]. Next, our data demonstrated that*C4A*copy numbers conferred a stronger risk reduction in females (OR 0.77 (0.68; 0.87)) compared to males (OR 0.91 (0.77; 1.06)), although the difference of observed effect sizes was not statistically significant (*P* > 0.05). Finally, we investigated the association in groups stratified by both, age and gender, and found that particularly females in the oldest age group (above 78 years) had the strongest protective effect of additional *C4A *copies (Additional file 6: Figure S2). Again, this effect was observed in a similar fashion across the three studies.

### C4A CNVs are associated with AMD independently of CFB/C2 risk variants

Previous work showed a strong association of four independent signals in the complement factor B/complement component 2 (*CFB/C2*) locus with AMD [9]. The *CFB/C2 *locus is in close proximity to the *C4 *gene on chromosome 6 which potentially could explain the observed association [27]. We therefore fitted multivariate logistic regression models conditioned on four SNPs at *CFB/C2 *(rs429608, rs114190211, rs204993, and rs142511358) and found a protective, although smaller and statistically no longer significant effect per copy number when adjusting for these variants (OR 0.93 (0.82; 1.06)). This loss of association was mainly driven by rs429608 (representing independent hit IH8.1 in Ref. [9]), as C4A copy number was significantly associated with AMD risk in a model incorporating only rs114190211, rs204993, and rs142511358 (OR 0.84 (0.75; 0.94)). Furthermore, the reduction in effect sizes appeared to be predominantly present in younger individuals and in males (Additional file 6: Figure S2). The association remained strong and significant in females (OR conditioned on *CFB/C2 *variants: 0.86 (0.74; 0.99)) and in individuals in the highest age group (above 78 years, OR conditioned on *CFB/C2 *variants: 0.75 (0.61; 0.93)). Females in the oldest age category (>78 years) showed a similar association of C4A copy number with AMD in the conditioned and in the unconditioned model (OR unconditioned on *CFB/C2 *variants: 0.62 (0.48; 0.79), OR conditioned on *CFB/C2 *variants: 0.68 (0.52; 0.90)). The independence of association in the latter group was observed across the three studies (OR of *C4A *copy number conditioned on *CFB/C2 *variants in females older than 78 years in the (1) MUE/TUE study: 0.74 (0.40; 1.34)), (2) WUE study: 0.71 (0.48; 1.03), and (3) AUS study: 0.55 (0.31; 0.94)).

### C4A CNVs are found mainly on two AMD-associated haplotypes in the CFB/C2 region

To further investigate the observed age and gender difference in the association of C4A CNVs, we used SHAPEIT2 to estimate the haplotypes in the CFB/C2 region. In total, we found six different haplotypes (termed H1 to H6) in this locus with an allele frequency above 1 % in the current study (Additional file 9: Figure S4). The average C4A copy number was lower on the risk increasing (adverse) haplotype H2 (characterized by the presence of the risk increasing allele at rs204993) and higher on the protective haplotype H3 (characterized by the presence of the risk reducing allele at rs429608). Both observations are consistent with the observed risk reduction with increasing C4A copy numbers. We therefore focused on the H2 and H3 haplotypes and investigated their impact on the association of C4A copy number. Adverse haplotype H2 only marginally altered the association of C4A CNV with AMD (OR C4A conditioned on H2: 0.86 (0.77; 0.97)), indicating that the association of C4A copy number with AMD risk is independent of haplotype H2. Similar to the results obtained from the single variant analysis, the haplotype H3 carrying the protective allele at rs429608 reduced the association of C4A CNV with AMD (OR C4A conditioned on H3: 0.92 (0.82; 1.03)). Consequently, we investigated the association of H3 with AMD risk stratified by age and gender (Additional file 9: Figure S4). While we failed to find a difference in the strength of the association between male and female, there was a correlation between increasing age and association strength. The association strength of the protective haplotype H3 decreased (less protective) with increasing age, in line with previous reports [30, 31]. Thus, it can be speculated that younger individuals are protected from AMD by variations found on protective haplotypes tagged by rs429608, while with age C4A copy numbers take over (have a stronger effect) and as such represent the main protective factor on those haplotypes.

## Discussion

Here, we performed a genetic association study of complement *C4*CNVs in 1536 AMD cases and 1109 unaffected controls from three independent studies [32]. This identified a strong protective association with an increase in copy numbers of the *C4A *isoform. *C4 *is known to play an important role in the activation of the classical and lectin pathways of the complement system [33, 34]. Although *C4A *and *C4B *share >99 % sequence identity, *C4A *has been suggested to have a primary role in immune complex clearance [26], greatly supported by the strong association between systemic lupus erythematosus (SLE) and low copy numbers of* C4A *[27, 28]. The involvement of immune complexes have also been reported to contribute to drusen formation [35], the earliest observable phenotypic changes in AMD etiology. Genomic copy numbers and serum C4A concentration are directly correlated [24, 36], and increasing plasma concentrations of C4A have been proposed to increase the clearance of immune complexes [37]. It can be speculated that accumulation of immune complexes are problematic in older individuals with reduced C4A, and as such could partially explain the observed increasing age-dependent association we report in this study.

The *C4 *gene is located on chromosome 6p21 in the MHC class III region around 30 kb proximal to the *CFB*/*C2 *locus known to be strongly associated with AMD [5, 38]. In SLE, physical proximity between *C4 *CNVs and variations at the *CFB*/*C2 *locus is thought to partially explain the observed association signals [27]. In contrast, in AMD, significant association of genes flanking the *C4 *locus were suspected independently of *CFB/C2 *variants [39, 40]. To further address the latter issue, we investigated the association of *C4A *with AMD risk after conditioning for four genetic variants known to carry the main signals at the *CFB/C2 *locus [9]. As a result, after conditioning, effect sizes and association strength of *C4A *copy numbers are reduced and no longer statistically significant. The loss of association is mainly driven by rs429608, as C4A copy number is significantly associated with AMD in a model conditioned on the remaining *CFB/C2 *variants, namely rs114190211, rs204993, and rs142511358. However, the association of *C4A *copy number with AMD in females and in individuals beyond 78 years of age remains statistically significant. Importantly, increased *C4A* copy number confers similar risk reduction in females of the age group >78 years in the *C2/CFB *conditioned versus the unconditioned model. This independence of association was observed across the three studies included in the analysis.

By analyzing AMD-associated haplotypes, we find *C4A* copy number to be correlated to two AMD-associated haplotypes in the *CFB/C2 *region (haplotypes H2 and H3). We show that the association of *C4A *copy number is independent of the adverse haplotype H2 but not of the protective haplotype H3, the latter tagged by rs429608. Importantly, we observed a strong reduction in the protective effect of H3 in older individuals, in line with previous results [30, 31]. These findings led us to hypothesize that upon reduced protective impact of the H3 haplotype on older individuals, C4A CNVs significantly influence disease risk in this age group. In contrast, in younger individuals, the protective effect of the H3 haplotype would be stronger and thus would account for most of the protection conferred by the C2/CFB locus in those individuals. With this in mind, our data support the notion that older individuals reveal the CNV at *C4A *to be associated with AMD risk independently of known risk variations at the *C2/CFB *locus.

So far, AMD pathogenesis has primarily been linked to dysregulation of the alternative pathway of the complement system [9]; however, expression analysis in cells of the RPE-choroid complex has also identified components and regulatory molecules associated with the classical pathway [41] and implicated classical complement activation in various retinal degenerations [42]. Moreover, the presence of immunoglobulin G (IgG) and terminal C5b-9 complement complexes as a component of drusen deposits are indicative of classical pathway activation [35, 43]. These data, together with our findings in this study, suggest the classical pathway, in addition to the alternative pathway, to play a causal role in pathological events leading to AMD disease.

The mechanisms underlying gender differences in AMD risk are still unknown. We recently reported that genetic variants in the death-associated protein-like 1 (*DAPL1*) gene are associated with increased risk for AMD, with risk association significantly higher in females than in males [17]. In the current study, we have identified another gender-specific locus at *C4A*, in this instance with a strong protective effect in females. Taken together, these data provide a reasonable basis for further investigations into the gender bias observed in AMD prevalence.

We considered age to possibly be a confounding factor in this study as previous reports have linked copy number of *C4B *and total *C4 *with longevity [44, 45]. However, in this study, we found no correlation between total *C4*, *C4A*, or *C4B* copy number and age when including all individuals in the calculations (*P* > 0.05). Additionally, our AMD association analyses were adjusted for age, which should satisfactorily account for confounding effects of longevity.

## Conclusions

In conclusion, our results demonstrate that multiallelic CNVs provide another source of genetic variance that need to be considered in complex diseases such as AMD. To date, an 84-kb deletion encompassing the *CFHR3*/*CFHR1 *genes [46] and a small complex insertion/deletion polymorphism in the *ARMS2 *gene [47] represent the only CNVs to be reproducibly associated with AMD disease. It is noteworthy that the *CFHR3*/*CFHR1 *CNV is also associated with SLE, suggesting that these two etiologies may share overlapping disease pathways. While our significance level does not reach current standards of genome-wide significance (~5.00 × 10^−8^) for new associated loci, it is questionable whether this threshold is overly conservative for complex CNVs such as *C4*, especially since we only investigated three mutually correlated CNVs. In addition, other replicated multiallelic CNV associations did not reach this threshold [48]. Our finding that increased copy number of *C4A *is associated with protection in AMD may have direct implications for therapy, as targeted approaches to molecular constituents of the complement pathway have the potential for early intervention before vision is compromised. This is especially true, in very contrast to most associated single nucleotide variants, as we can directly implicate the relevant gene (*C4A*) and the orientation of the protective effect with increased copy numbers and thus with increased C4A protein levels in serum [49].

## Additional files

Additional file 1: Table S1. List of custom made oligonucleotide probes for MLPA genotyping. (DOCX 15 kb) Additional file 2: Figure S1. Correlation between total *C4 *dosage and *C4A/C4B *dosage in the WUE and MUE/TUE studies. Total C4 dosage is highly correlated to the mean of C4A and C4B dosages in the WUE and MUE/TUE studies (linear regression slop = 0.914; R^2^ = 0.895). The MLPA based dosages show predominant clustering of measurements most frequently centered around integer values 2,3,4,5 and 6 representing the respective total C4 copy numbers. Consequently, for the AUS study total C4 dosage can be estimated by simply calculating the mean of C4A and C4B dosage. (TIF 2198 kb) Additional file 3: Table S2. Frequency and percentages of each *C4 *copy number subtype and total *C4 *in cases and controls. (DOCX 14 kb) Additional file 4: Table S3. Isotype specific primers for confirmation of homozygous deletions in *C4A *and *C4B. (DOCX 13 kb)*
Additional file 5: Table S4. Sensitivity analysis for C4A copy number association. (DOCX 17 kb) Additional file 6: Figure S2. Subgroup/sensitivity analysis in the pooled study of *C4A *copy number. Odds ratios and corresponding 95 % confidence intervals are given with the size of each rectangle representing the respective relative number of cases and controls in each subgroup. The protective effect of increasing *C4A *copy number is stronger in females and increases with age. Both effects can also be observed when conditioning on known AMD associated risk variants at the *C2/CFB *locus (rs429608, rs114190211, rs204993 and rs142511358 [9]) and are present in each of the individual studies. 95 % confidence intervals are indicated; N stands for the total number of individuals included in the analysis. (TIF 1946 kb) Additional file 7: Figure S3. Sensitivity analysis for protective haplotype H3 at the C2/CFB locus. Phase at C2/CFB was assessed with SHAPEIT2 for each individual. Haplotypes are characterized by the presence or absence of AMD associated alleles from four inpendent variations at this locus (rs429608, rs114190211, rs204993 and rs142511358 [9]). Odds ratios and corresponding 95 % confidence intervals [95 % CI] are given with the size of each rectangle representing the respective relative number of cases and controls for each subgroup. The protective effect of haplotype H3 decreases with age (becomes less protective). (TIF 1225 kb) Additional file 8: Table S5. Mean dosages (S.D.) for diploid and tetraploid cases and controls for *C4A*, *C4B *and total*C4. (DOCX 14 kb)*
Additional file 9: Figure S4. AMD associated haplotypes at the C2/CFB locus on chromosome 6. The phase at C2/CFB was assessed with SHAPEIT2 for each individual. In total, we found six haplotypes (H1-H6) with an allele frequency ≥ 1 % in the study. The haplotypes are characterized by the presence or absence of AMD associated alleles from four inpendent variations at this locus (rs429608, rs114190211, rs204993 and rs142511358 [9]). C4A CNVs are predominantly present on the adverse haplotype H2 (carrying the G allele of rs204993) and the protective haplotype H3 (carrying the A allele of rs429608). OR = Odds ratio, [95 % CI] = 95 % confidence intervals, SD = standard deviation. The haplotypes were numbered according to their frequency. (TIF 2488 kb)
