# Supplementary material for: Multiallelic copy number variation in the complement component 4A (C4A) gene is associated with late-stage age-related macular degeneration (AMD)
Source: J Neuroinflammation. 2016 Apr 18;13:81. doi: 10.1186/s12974-016-0548-0 (PMC4835888; doi:10.1186/s12974-016-0548-0)
Supplement: Additional file 1: Table S1. — List of custom made oligonucleotide probes for MLPA genotyping. (DOCX 15 kb) [file 12974_2016_548_MOESM1_ESM.docx]

| Gene ID | Left Hybridising Sequence 5'-3' | Right Hybridising Sequence 3'-5' |
| --- | --- | --- |
| C4B | GGGTTCCCTAAGGGTTGGACTCGTTCCAGGACCTCTCTCCAGTGA | TACATAGGAGCATGCAGGTGCGGGTCTAGATTGGATCTTGCTGGC |
| C4A | GGGTTCCCTAAGGGTTGGACTGACTCGTTCCAGGACCCCTGTCCAGTGT | TAGACAGGAGCATGCAGGTGCGGGTCTAGATTGGATCTTGCTGGC |
| C4-ex 3 | GGGTTCCCTAAGGGTTGGAGAACGACAAACATCCAGGGTATCAACCTGCTCTTCTCCTCTCGCCGG | GGGCACCTCTTTTTGCAGACGGACCAGCCCATTTACAACCCTGGCCAGCTCTATCTAGATTGGATCTTGCTGGC |
| C4-ex30 | GGGTTCCCTAAGGGTTGGAGTCTCAATGTGACTCTCAGCTCCACAGGCCGG | AATGGGTTCAAGTCCCACGCGCTGCAGCTGAACAACTCTAGATTGGATCTTGCTGGC |
| CREBBP_1 (con) | GGGTTCCCTAAGGGTTGGACCAGCTAGTGGAATTCAAAACACAATTGGTTCTGTTGGCACA | GGGCAACAGAATGCCACTTCTTTAAGTAACTCTAGATTGGATCTTGCTGGC |
| EP300_1 (con) | GGGTTCCCTAAGGGTTGGACCAACCTAAGCACTGTTAGTCAGATTGATCCCAGCTCCAT | AGAAAGAGCCTATGCAGCTCTTGGACTACCCTATCATCTAGATTGGATCTTGCTGGC |
| MLPA_1 | GGGTTCCCTAAGGGTTGGACCATCATGCGTATGCCTGATGAGCTGATACCCAATGGAGGAGAAT | TAGGCCTTTTAAACAGTGGGAACCTTGTTCCAGTCTAGATTGGATCTTGCTGGC |
| MLPA_2 | GGGTTCCCTAAGGGTTGGAGGGACTAACCAATGGTGGTGATATTAATCAGCT | TCAGACAAGTCTTGGCATGGTACAAGATGTCTAGATTGGATCTTGCTGGC |
| MLPA_3 | GGGTTCCCTAAGGGTTGGACAGTAGCCGGCTGCTGGCATGCCATCTCTCCA | GCACACGACACCACCTGGGATGACTCTAGATTGGATCTTGCTGGCGC |
| MLPA_4 | GGGTTCCCTAAGGGTTGGACGTACCCTGCCTCTTCTCATTACCTCTCATGCA | CCATCGTAGGGATCATAGTTCTAATTGTGCTTCTCTAGATTGGATCTTGCTGGC |

**Supplementary Table 1.** List of custom made oligonucleotide probes for MLPA genotyping
