# Supplementary figures and images for: Multiallelic copy number variation in the complement component 4A (C4A) gene is associated with late-stage age-related macular degeneration (AMD)
Source: J Neuroinflammation. 2016 Apr 18;13:81. doi: 10.1186/s12974-016-0548-0 (PMC4835888; doi:10.1186/s12974-016-0548-0)

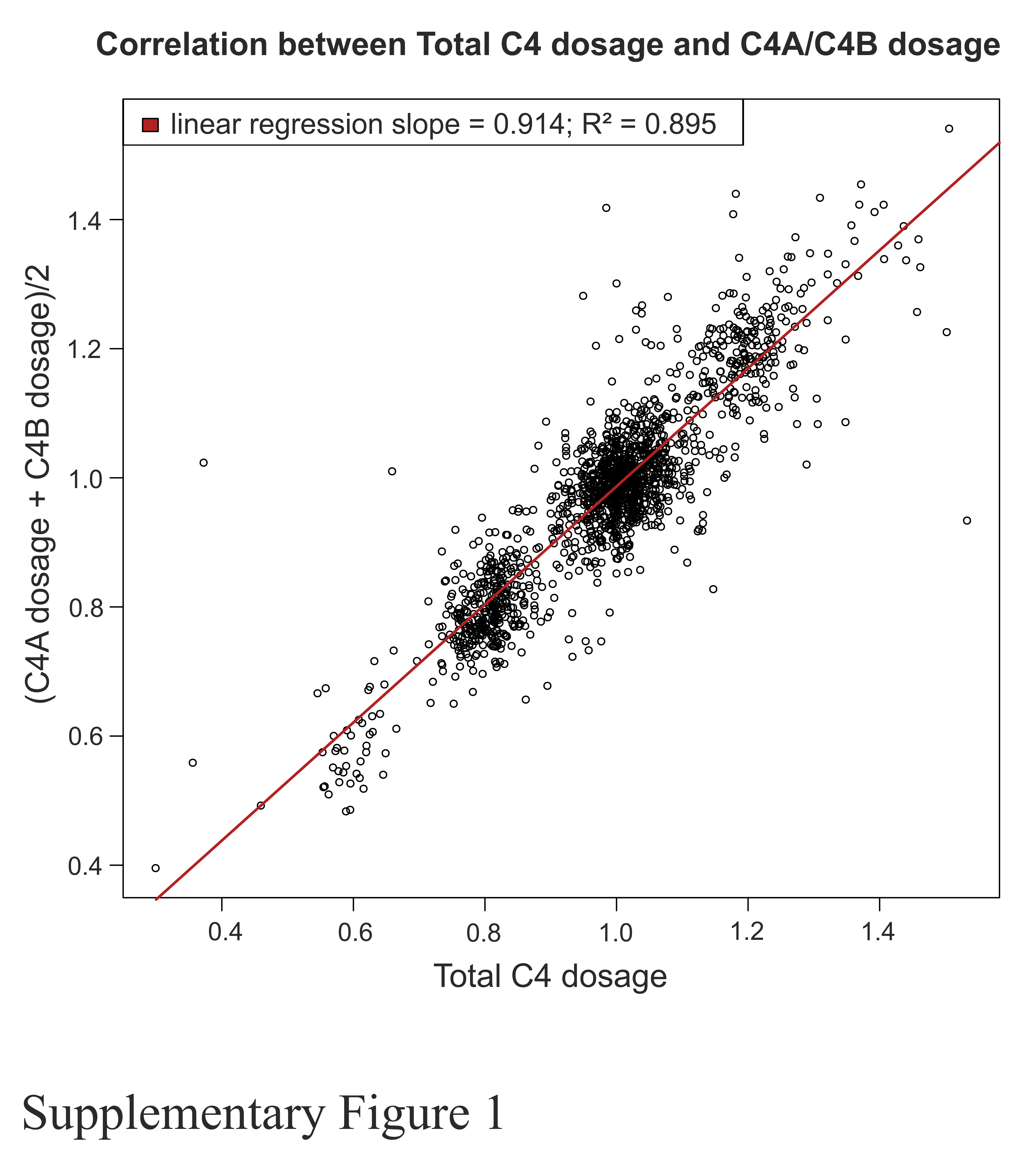

Supplement: Additional file 2: Figure S1. — Correlation between total C4 dosage and C4A/C4B dosage in the WUE and MUE/TUE studies. Total C4 dosage is highly correlated to the mean of C4A and C4B dosages in the WUE and MUE/TUE studies (linear regression slop = 0.914; R2 = 0.895). The MLPA based dosages show predominant clustering of measurements most frequently centered around integer values 2,3,4,5 and 6 representing the respective total C4 copy numbers. Consequently, for the AUS study total C4 dosage can be estimated by simply calculating the mean of C4A and C4B dosage. (TIF 2198 kb) [file 12974_2016_548_MOESM2_ESM.tif]

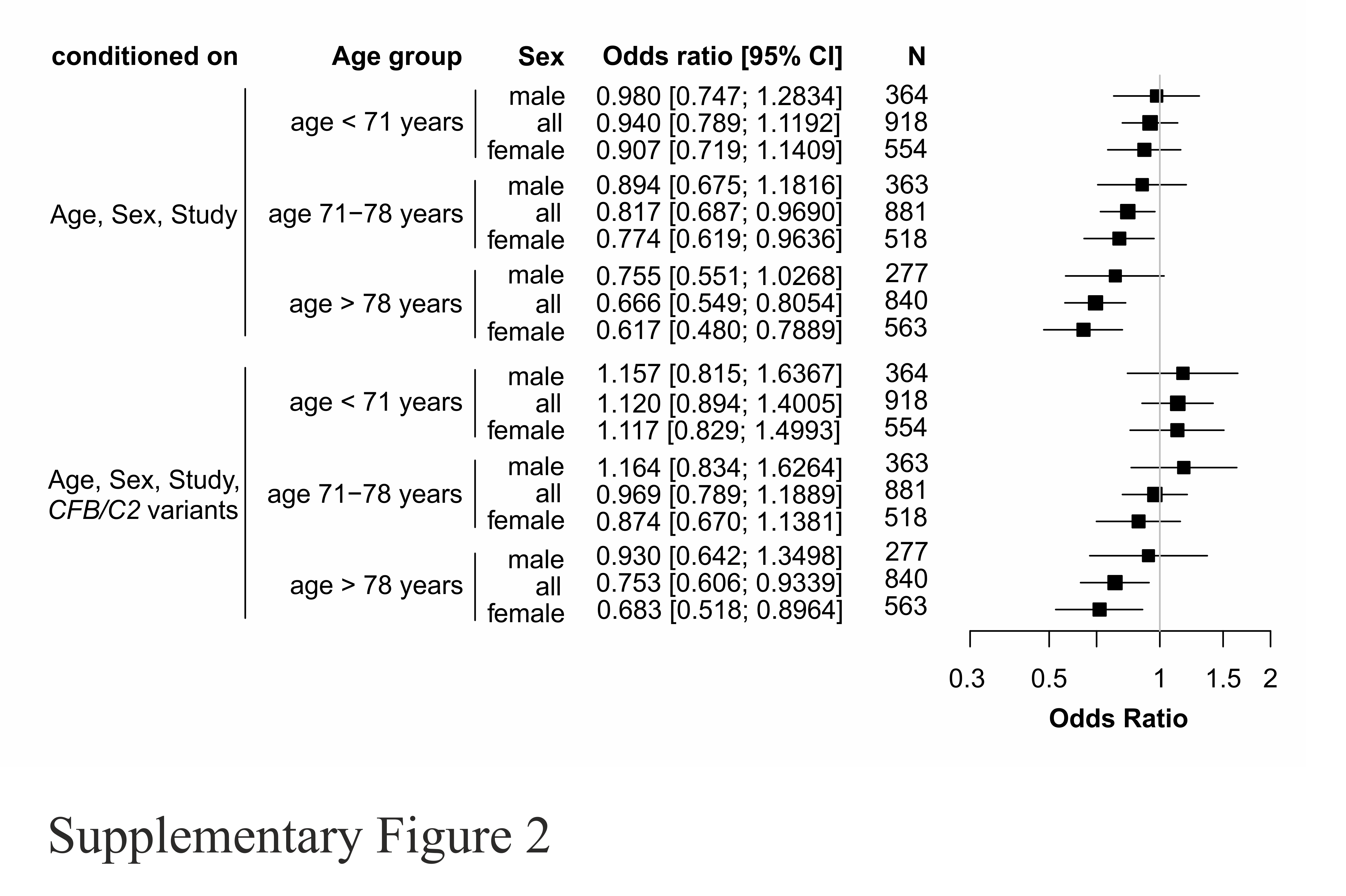

Supplement: Additional file 6: Figure S2. — Subgroup/sensitivity analysis in the pooled study of C4A copy number. Odds ratios and corresponding 95 % confidence intervals are given with the size of each rectangle representing the respective relative number of cases and controls in each subgroup. The protective effect of increasing C4A copy number is stronger in females and increases with age. Both effects can also be observed when conditioning on known AMD associated risk variants at the C2/CFB locus (rs429608, rs114190211, rs204993 and rs142511358 [9]) and are present in each of the individual studies. 95 % confidence intervals are indicated; N stands for the total number of individuals included in the analysis. (TIF 1946 kb) [file 12974_2016_548_MOESM6_ESM.tif]

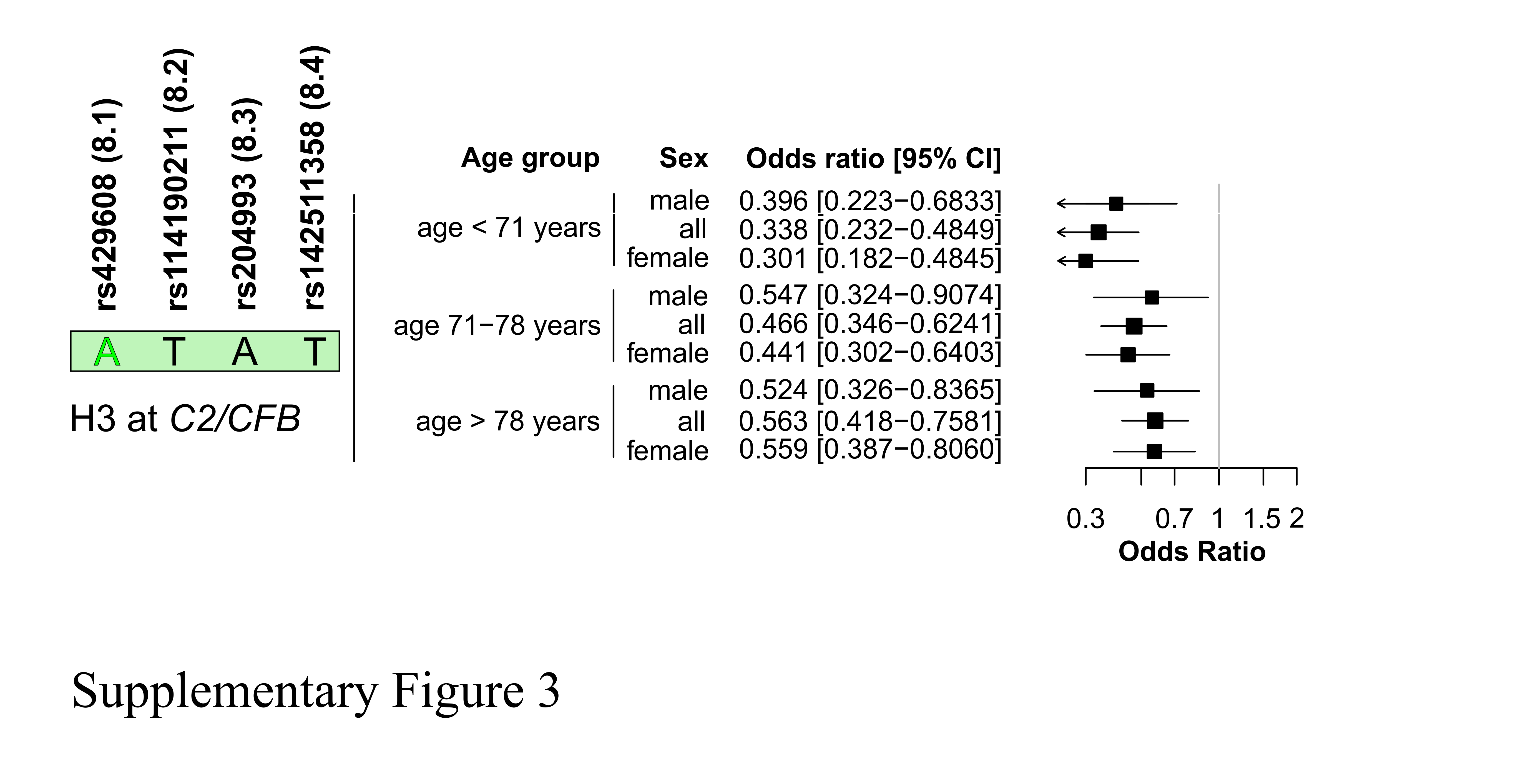

Supplement: Additional file 7: Figure S3. — Sensitivity analysis for protective haplotype H3 at the C2/CFB locus. Phase at C2/CFB was assessed with SHAPEIT2 for each individual. Haplotypes are characterized by the presence or absence of AMD associated alleles from four inpendent variations at this locus (rs429608, rs114190211, rs204993 and rs142511358 [9]). Odds ratios and corresponding 95 % confidence intervals [95 % CI] are given with the size of each rectangle representing the respective relative number of cases and controls for each subgroup. The protective effect of haplotype H3 decreases with age (becomes less protective). (TIF 1225 kb) [file 12974_2016_548_MOESM7_ESM.tif]

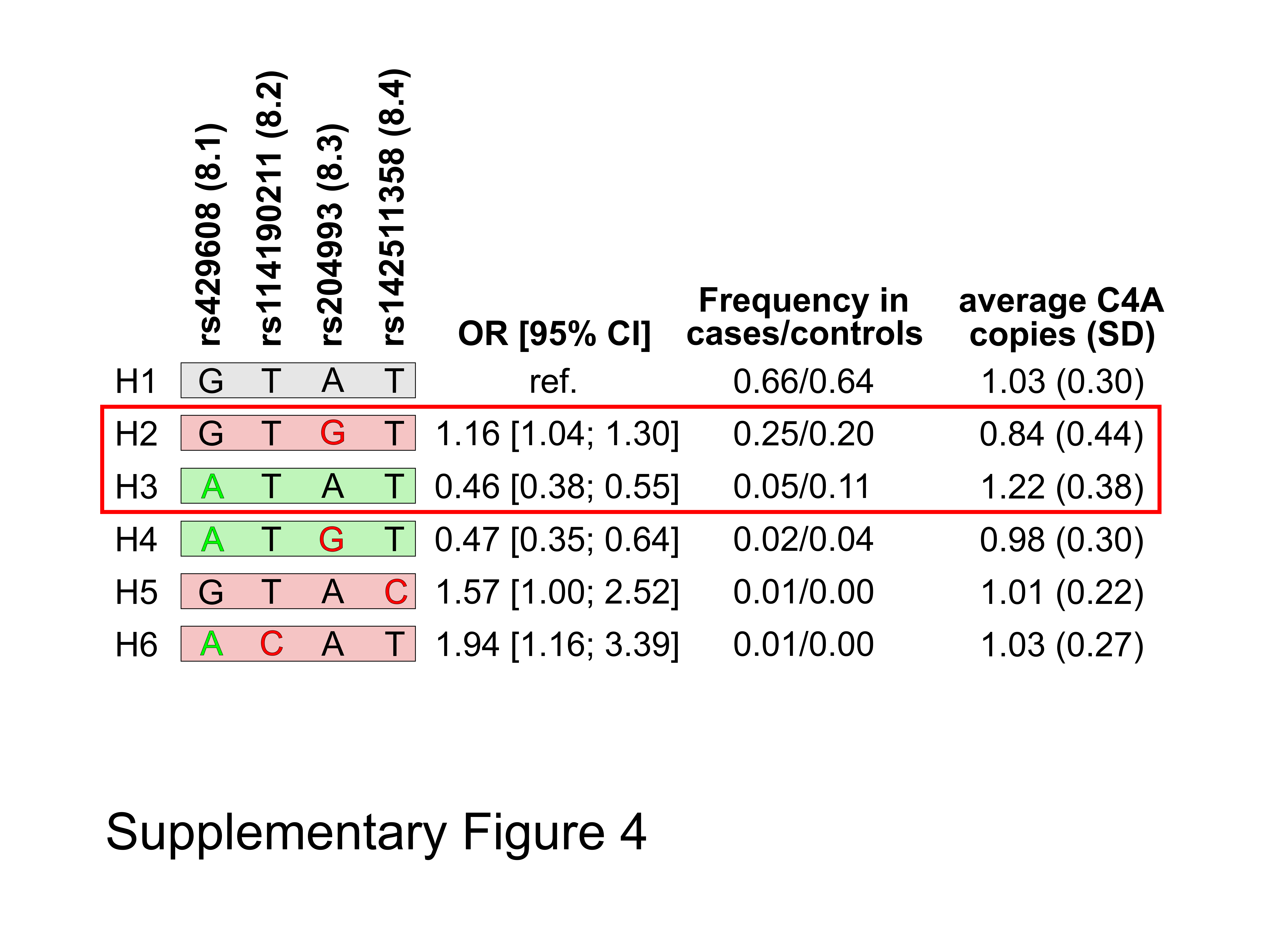

Supplement: Additional file 9: Figure S4. — AMD associated haplotypes at the C2/CFB locus on chromosome 6. The phase at C2/CFB was assessed with SHAPEIT2 for each individual. In total, we found six haplotypes (H1-H6) with an allele frequency ≥ 1 % in the study. The haplotypes are characterized by the presence or absence of AMD associated alleles from four inpendent variations at this locus (rs429608, rs114190211, rs204993 and rs142511358 [9]). C4A CNVs are predominantly present on the adverse haplotype H2 (carrying the G allele of rs204993) and the protective haplotype H3 (carrying the A allele of rs429608). OR = Odds ratio, [95 % CI] = 95 % confidence intervals, SD = standard deviation. The haplotypes were numbered according to their frequency. (TIF 2488 kb) [file 12974_2016_548_MOESM9_ESM.tif]
