# Supplementary material for: Multiallelic copy number variation in the complement component 4A (C4A) gene is associated with late-stage age-related macular degeneration (AMD)
Source: J Neuroinflammation. 2016 Apr 18;13:81. doi: 10.1186/s12974-016-0548-0 (PMC4835888; doi:10.1186/s12974-016-0548-0)
Supplement: Additional file 3: Table S2. — Frequency and percentages of each C4 copy number subtype and total C4 in cases and controls. (DOCX 14 kb) [file 12974_2016_548_MOESM3_ESM.docx]

**Supplementary Table 2.** Frequency and percentages of each *C4* copy number subtype and total *C4* in cases and controls

|  | Copy number (%) | | | | | | | |
| --- | --- | --- | --- | --- | --- | --- | --- | --- |
|  | 0 | 1 | 2 | 3 | 4 | 5 | 6 | 7 |
| *C4A* Cases | 36 (2.4) | 282 (18.4) | 867 (56.6) | 291 (19.0) | 51 (3.3) | 5 (0.3) | 0 | 0 |
| *C4A* Controls | 16 (1.4) | 176 (15.9) | 613 (55.3) | 236 (21.3) | 62 (5.6) | 6 (0.5) | 0 | 0 |
| *C4A* UK Controls [27] | 9 (1.25) | 149 (20.7) | 375 (52.2) | 143 (19.9) | 43 (5.9) | 0 | 0 | 0 |
| *C4A* EU Controls [28] | 5 (0.97) | 89 (17.4) | 289 (56.3) | 111 (21.6) | 17 (3.3) | 2 (0.4) | 0 | 0 |
| *C4B* Cases | 41 (2.7) | 365 (23.8) | 102 (66.5) | 102 (6.6) | 6 (0.4) | 1 (0.1) | 0 | 0 |
| *C4B* Controls | 25 (2.3) | 296 (26.7) | 702 (63.4) | 80 (7.2) | 4 (0.4) | 0 | 0 | 0 |
| *C4B* UK Controls [27] | 26 (3.6) | 185 (25.7) | 410 (57.0) | 94 (13.0) | 4 (0.6) | 0 | 0 | 0 |
| *C4B* EU Controls [28] | 14 (2.7) | 138 (27.0) | 325 (63.5) | 35 (6.8) | 1 (0.2) | 0 | 0 | 0 |
| Total *C4* Cases | 0 | 0 | 49 (3.2) | 398 (26.0) | 885 (57.8) | 178 (11.6) | 21 (1.4) | 1 (0.1) |
| Total *C4* Controls | 0 | 0 | 29 (2.6) | 244 (22.0) | 632 (57.1) | 187 (16.9) | 14 (1.3) | 1 (0.1) |
| Total *C4* UK Controls [27] | 0 | 0 | 25 (3.5) | 168 (23.4) | 385 (53.0) | 137 (19.0) | 4 (0.6) | 0 |
| Total *C4* EU Controls [28] | 0 | 0 | 7 (1.4) | 135 (26.1) | 312 (60.4) | 53 (10.3) | 10 (1.9) | 0 |
