# Supplementary material for: Multiallelic copy number variation in the complement component 4A (C4A) gene is associated with late-stage age-related macular degeneration (AMD)
Source: J Neuroinflammation. 2016 Apr 18;13:81. doi: 10.1186/s12974-016-0548-0 (PMC4835888; doi:10.1186/s12974-016-0548-0)
Supplement: Additional file 4: Table S3. — Isotype specific primers for confirmation of homozygous deletions in C4A and C4B. (DOCX 13 kb) [file 12974_2016_548_MOESM4_ESM.docx]

**Supplementary Table 3**. Isotype specific primers for confirmation of homozygous deletions in *C4A* and *C4B*

| **Primer Names** | **Sequences 5’-3’** |
| --- | --- |
| *C4A* up-F | GCATGCTCCTGTCTAACACTGGAC |
| *C4B* up-F | TGCTCCTATGTATCACTGGAGAGA |
| L3_R | TGCGGATCCAGCAGTTTCGGAAG |
| *C4A* down- F | AGGACCCCTGTCCAGTGTTAGAC |
| *C4B* down- F | AGGACCTCTCTCCAGTGATACAT |
| L4_R | ATAGGATCCTAAGGTCCCCTGGGCCT |
