# Supplementary material for: Multiallelic copy number variation in the complement component 4A (C4A) gene is associated with late-stage age-related macular degeneration (AMD)
Source: J Neuroinflammation. 2016 Apr 18;13:81. doi: 10.1186/s12974-016-0548-0 (PMC4835888; doi:10.1186/s12974-016-0548-0)
Supplement: Additional file 5: Table S4. — Sensitivity analysis for C4A copy number association. (DOCX 17 kb) [file 12974_2016_548_MOESM5_ESM.docx]

**Supplementary Table 4.** Sensitivity analysis for C4A copy number association

|  |  |  |  | Mean CN^1^ in | |  | Number of | |
| --- | --- | --- | --- | --- | --- | --- | --- | --- |
| Analysis | P-value | OR per CN^1^ | 95%CI | Controls | Cases |  | Controls | Cases |
| unadjusted | 0.00022 | 0.83 | [0.76; 0.92] | 2.15 | 2.04 |  | 1109 | 1536 |
| adjusted* | 7.11x10^-05^ | 0.82 | [0.74; 0.90] | 2.15 | 2.04 |  | 1109 | 1536 |
| age < 71 y* | 0.49 | 0.94 | [0.79; 1.12] | 2.09 | 2.04 |  | 456 | 462 |
| age 71-78 y* | 0.02 | 0.82 | [0.74; 0.90] | 2.17 | 2.05 |  | 367 | 514 |
| age > 78 y* | 3.2 x10^-05^ | 0.67 | [0.55; 0.81] | 2.24 | 2.01 |  | 283 | 557 |
| male # | 0.219 | 0.91 | [0.77; 1.06] | 2.13 | 2.07 |  | 462 | 543 |
| female # | 4.87 x10^-05^ | 0.77 | [0.68; 0.87] | 2.18 | 2.02 |  | 645 | 990 |
| GA*^2^ | 0.00360 | 0.81 | [0.71; 0.93] | 2.15 | 2.03 |  | 1109 | 454 |
| NV*^3^ | 0.00040 | 0.82 | [0.73; 0.91] | 2.15 | 2.04 |  | 1109 | 912 |
| GA&NV*^4^ | 0.02277 | 0.75 | [0.59; 0.96] | 2.15 | 2.03 |  | 1109 | 170 |

*adjusted for Age, Sex and Study

# adjusted for Age and Study

^1^ CN = copy number

^2^ patients with geographic atrophy compared to controls

^3^ patients with neovascular AMD compared to controls

^4^ both late stage forms [GA&NV] compared to controls.
