# Supplementary material for: Multiallelic copy number variation in the complement component 4A (C4A) gene is associated with late-stage age-related macular degeneration (AMD)
Source: J Neuroinflammation. 2016 Apr 18;13:81. doi: 10.1186/s12974-016-0548-0 (PMC4835888; doi:10.1186/s12974-016-0548-0)
Supplement: Additional file 8: Table S5. — Mean dosages (S.D.) for diploid and tetraploid cases and controls for C4A, C4B and totalC4. (DOCX 14 kb) [file 12974_2016_548_MOESM8_ESM.docx]

| **Supplementary Table 5.** Mean dosages (S.D.) for diploid and tetraploid cases and controls for *C4A*, *C4B* and total *C4* | | | | | |
| --- | --- | --- | --- | --- | --- |
|  |  | ALL | AUS | WUE | MUE/TUE |
| C4A | cases, diploid | 0.993 (0.066) | 0.988 (0.085) | 0.994 (0.052) | 0.998 (0.05) |
|  | controls, diploid | 1.005 (0.069) | 1.017 (0.094) | 1.004 (0.047) | 0.991 (0.052) |
| C4B | cases, diploid | 1.034 (0.074) | 1.036 (0.066) | 1.034 (0.079) | 1.029 (0.077) |
|  | controls, diploid | 1.025 (0.072) | 1.021 (0.061) | 1.03 (0.077) | 1.025 (0.079) |
| Total C4 | cases, tetraploid | 1.007 (0.058) | 1.005 (0.059) | 1.008 (0.045) | 1.008 (0.071) |
|  | controls, tetraploid | 1.012 (0.057) | 1.01 (0.057) | 1.015 (0.042) | 1.009 (0.072) |
